# Enhanced Glutathione Content Allows the In Vivo Synthesis of Fluorescent CdTe Nanoparticles by *Escherichia coli*

**DOI:** 10.1371/journal.pone.0048657

**Published:** 2012-11-21

**Authors:** Juan P. Monrás, Víctor Díaz, Denisse Bravo, Rebecca A. Montes, Thomas G. Chasteen, Igor O. Osorio-Román, Claudio C. Vásquez, José M. Pérez-Donoso

**Affiliations:** 1 Microbiology and Bionanotechnology Research Group, Laboratorio de Bioquímica, Departamento de Bioquímica y Biología Molecular, Facultad de Ciencias Químicas y Farmacéuticas, Universidad de Chile, Santiago, Chile; 2 Laboratorio de Microbiología Molecular, Departamento de Biología, Universidad de Santiago de Chile, Santiago, Chile; 3 Laboratorio de Microbiología Oral, Facultad de Odontología, Universidad de Chile, Santiago, Chile; 4 Department of Chemistry, Sam Houston State University, Huntsville, Texas, United States of America; 5 Departamento de Química Inorgánica, Pontificia Universidad Católica de Chile, Santiago, Chile; 6 Departamento de Ciencias Biológicas, Universidad Andrés Bello, Santiago, Chile; RMIT University, Australia

## Abstract

The vast application of fluorescent semiconductor nanoparticles (NPs) or quantum dots (QDs) has prompted the development of new, cheap and safer methods that allow generating QDs with improved biocompatibility. In this context, green or biological QDs production represents a still unexplored area. This work reports the intracellular CdTe QDs biosynthesis in bacteria. *Escherichia coli* overexpressing the *gshA* gene, involved in glutathione (GSH) biosynthesis, was used to produce CdTe QDs. Cells exhibited higher reduced thiols, GSH and Cd/Te contents that allow generating fluorescent intracellular NP-like structures when exposed to CdCl_2_ and K_2_TeO_3_. Fluorescence microscopy revealed that QDs-producing cells accumulate defined structures of various colors, suggesting the production of differently-sized NPs. Purified fluorescent NPs exhibited structural and spectroscopic properties characteristic of CdTe QDs, as size and absorption/emission spectra. Elemental analysis confirmed that biosynthesized QDs were formed by Cd and Te with Cd/Te ratios expected for CdTe QDs. Finally, fluorescent properties of QDs-producing cells, such as color and intensity, were improved by temperature control and the use of reducing buffers.

## Introduction

Semiconductor nanoparticles (NPs) or quantum dots (QDs) are bimetallic structures of elements like Cd, S, Se or Te that given their particular physicochemical and optoelectronic properties exhibit great technological potential. These semiconductor NPs have the ability of absorbing light and emitting the exceeding energy in the form of fluorescence at high yields. The absorption and emission spectra of QDs depend on size and shape of the nanoparticle due to a quantum confinement effect. The idea of slightly changing the shape of these NPs and hence their optical properties has made them very popular in optoelectronics. In particular, CdTe QDs are used in electronic and optoelectronic devices and during the last decade, as an important tool for new solar cell technology (photovoltaic panels) and in biomedicine [1], [2].

Currently, CdTe QDs synthesis involves chemical procedures requiring high temperature, anaerobic conditions, toxic substrates/residues and displays unfavorable capital/energy ratios. As a consequence, QDs produced by these methods involve organic solvents and the resulting nanoparticles are not capped with soluble, more compatible agents. This renders chemically synthesized Quantum dots difficult to work with because of its insolubility and elevated citotoxicity due to cadmium, thus decreasing their potential applications. Development of new, simpler, less toxic and environmentally friendly synthesis procedures is then a subject of growing interest.

Metal-bacteria interactions play a crucial role in a number of biotechnological applications [3]. Microorganisms in general and bacteria in particular, are able to produce different metallic nanoparticles as gold, silver or iron particles, among others [4]–[6]. During the last decade, some advances were made regarding the mechanism underlying the synthesis of iron NPs by magnetotactic bacteria [7], [8]. These bacteria synthesize intracellular magnetic nanocrystals consisting of Fe_3_O_4_ or Fe_3_S_4_ (35–120 nm) that are surrounded by a membranous structure known as magnetosome [7]. Although some work has been dedicated to the control of NP shape and size in biologically synthesized silver and gold nanoparticles [9], [10], the molecular functions that govern this processes are still unknown.

A few reports regarding the use of microorganisms for synthesizing fluorescent semiconductor NPs have been published to date. Some experiments related to CdS QDs synthesis using *E. coli* and the yeast *Schizosaccharomyces pombe* have been reported [11], [12]. Also, extracellular CdSe biosynthesis using *Fusarium oxysporum* or *Saccharomyces cerevisiae* extracts has been recently communicated [13], [14]. The importance of metal-binding peptides in the biosynthesis process was established in all these communications. For example, the *Arabidopsis thaliana* protein pythochelatin was used for QDs synthesis in *E. coli* and *S. cerevisiae* [11], [14]. Recently, the use of *E. coli* overexpressing a specific histidine-rich peptide with affinity for CdS that facilitates the intracellular synthesis of CdS QDs was reported [15].

Recently, Bao *et al*. [16], [17] reported the extracellular synthesis of CdTe QDs by *E. coli* and *S. cerevisiae*. CdTe synthesis occurred in LB rich media and the presence of cells, cadmium and tellurium salts (CdCl_2_, Na_2_TeO_3_) and the strong chemical reducer NaBH_4_ was required. Interestingly, when compared to chemically-produced CdTe QDs, *S. cerevisiae*-biosynthesized QDs exhibited increased biocompatibility in HeLa cells [16], [17]. Also, during 2010 Park *et al*. [18] reported the intracellular synthesis of CdTe and other semiconductor NPs by overexpressing heterologous metal-binding proteins such as metallothionein or phytochelatin in *E. coli*.

Cellular events underlying the molecular mechanism(s) involved in bacterial production of QDs are still unknown and optimal conditions favoring CdTe QDs biosynthesis have not been determined to date. These considerations, in conjunction with unveiling biosynthetic mechanisms should allow the design of NPs with defined properties. In this context and to establish the basis for the biological synthesis of CdTe QDs, a procedure mimicking biological conditions was developed for the chemical synthesis of CdTe NPs [19]. The method requires just CdCl_2_, K_2_TeO_3_ and GSH as substrates and capping/reducing agent. Highly fluorescent QDs were obtained at temperatures, pH values and oxygen conditions similar to those found in microbial cells.

Taking into consideration GSH i) redox properties, ii) its abundance in cells and iii) interaction with K_2_TeO_3_ and CdCl_2_ [20]–[24], this work describes, for the first time, using this important biological thiol for intracellular CdTe QDs production by bacteria.

## Results

### *E. coli* GSH Content Assessment

**Figure 1:**
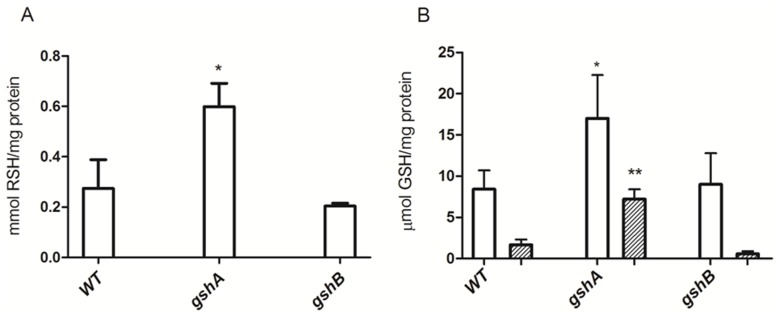
Total RSH and GSH content in *E. coli* overexpressing GSH biosynthesis genes. Cellular RSH (after 4 h IPTG induction, A) and GSH levels (B) after 4 h (white) or 16 h (striped) IPTG induction were determined in *E. coli* AG1 (wt), AG1/pCA24N*gshA* (*gshA*) and AG1/pCA24NgshB (*gshB*) cells (n = 3). *p<0.05; **p<0.005.

Since GSH is both a stabilizing and reducing agent during the chemical synthesis of CdTe QDs [19], we hypothesized that a bacterial strain containing increased levels of this tripeptide should be a good candidate for biosynthesizing this kind of NPs. To enhance GSH concentration, genes encoding the principal enzymes involved in glutathione biosynthesis, *gshA* and *gshB* [25], [26], were overexpressed in *E. coli*. Total RSH content was first determined in AG1, AG1/pCA24N*gshA* and AG1/pCA24N*gshB* strains (Fig. 1A). Despite a maximum expression level of GshA and GshB was observed after 4 h IPTG induction (Fig. S1), increased levels of cellular RSH were only observed in the *gshA*-expressing strain (Fig. 1A). To assess if it was paralleled by GSH overproduction, the intracellular GSH content was determined. As expected, GSH concentration increased in *E. coli* AG1/pCA24N*gshA*, but not in wild type or *gshB*-expressing cells (Fig. 1B).

Given that increased nanocrystal production has been observed in some microorganisms during stationary growth phase [27], [28], the GSH content was assessed during late stationary phase (after 16 h of IPTG induction). After this time, the GSH content in the *gshA*-expressing strain decreased as compared to that observed after 4 h induction (Fig. 1B). In the light of these results, the 4 h-induced AG1/pCA24N*gshA* strain represents the best choice to carry out QDs biosynthesis.

### Nanoparticle Biosynthesis

**Figure 2:**
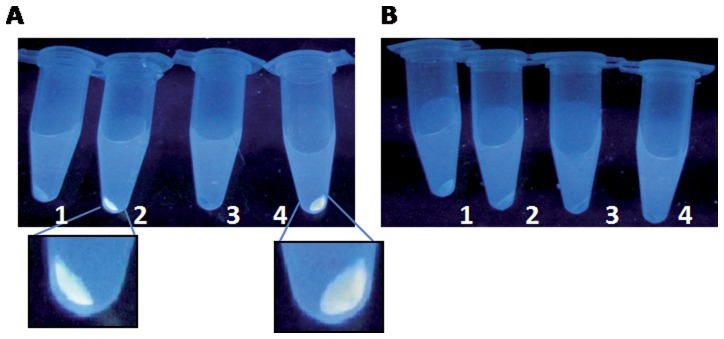
Fluorescence assessment in *E. coli* overexpressing *gshA* or *gshB* genes. UV light-exposed cell pellets of *E. coli* AG1/pCA24N*gshA* (A) or AG1/pCA24N*gshB* (B) that were untreated (1) or exposed to CdCl_2_ (2), K_2_TeO_3_ (3) or CdCl_2_/K_2_TeO_3_ (4).

**Figure 3:**
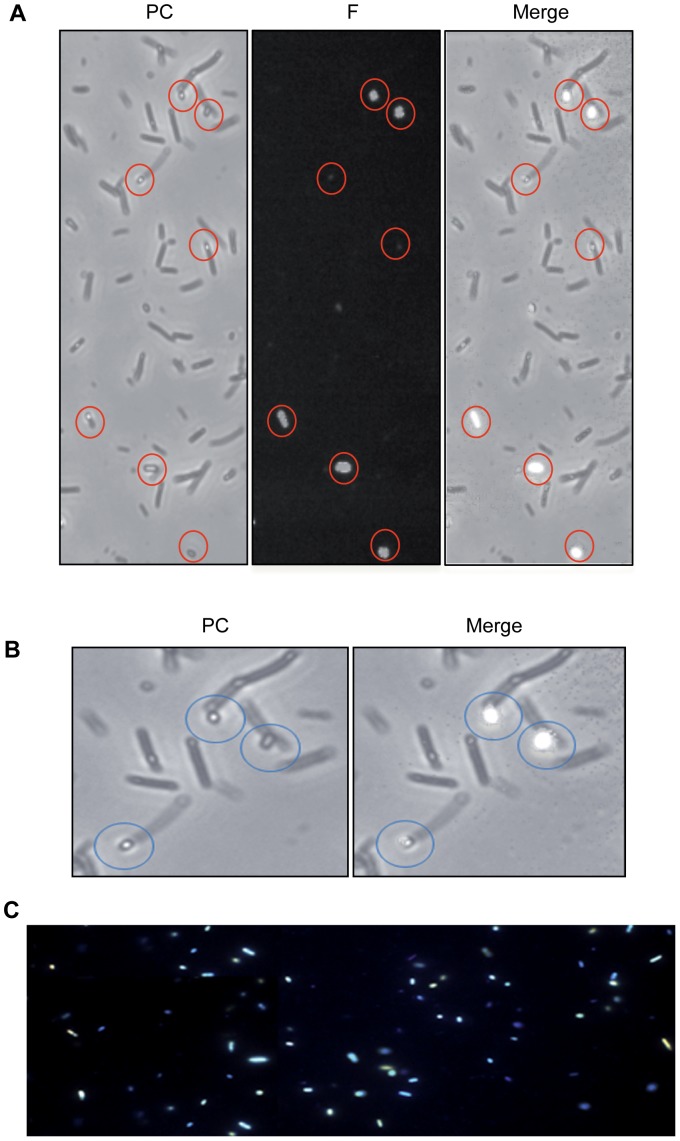
Fluorescence microscopy of *E. coli* exposed to Cd and Te salts. *E. coli* AG1/pCA24N*gshA* treated with CdCl_2_ and K_2_TeO_3_ was analyzed by epifluorescence microscopy. Circles indicate structures where fluorescence is accumulated. A, left image: phase contrast (PC); central image: monochromatic fluorescence (F) after excitation using UV-DAPI/FITC filter; right image: merge. B, left image: magnification phase contrast (PC); left image: merge. C, fluorescence microscopy under UV light exposure.

**Figure 4:**
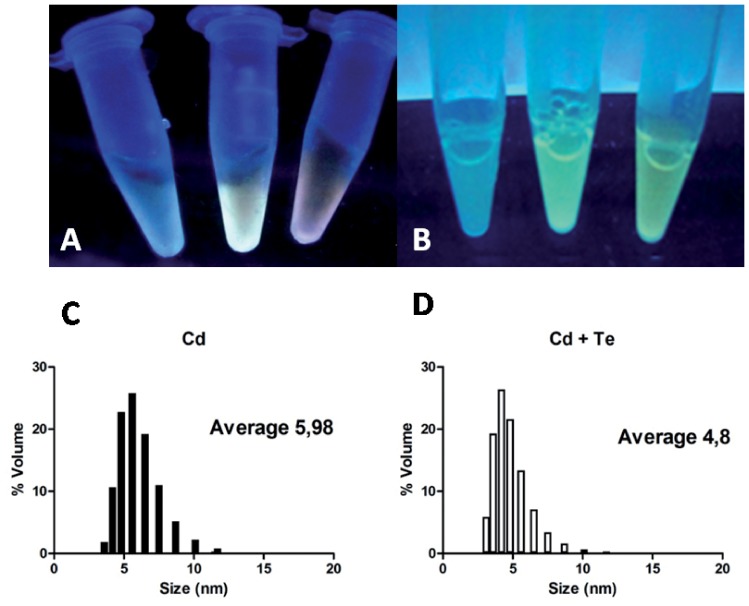
Particle purification and size determination. **A,** UV-exposed cell suspensions of *E. coli* AG1/pCA24N*gshA*, untreated or exposed to CdCl_2_ or CdCl_2_/K_2_TeO_3_ (from left to right). **B,** purified fractions exposed to UV light. **C** and **D**, DLS particle size determination of fluorescent samples from cells exposed to CdCl_2_ or CdCl_2_/K_2_TeO_3_, respectively.

Biosynthesis of fluorescent, semiconductor NPs was monitored in *E. coli* AG1/pCA24N*gshA* and AG1/pCA24N*gshB* strains as described in Methods. Given the intrinsic fluorescence properties of CdTe QDs, cell-based NP biosynthesis was assessed by exposing cell pellets, after metal treatment, to UV light (Fig. 2). Fluorescence was only observed in CdCl_2_- and CdCl_2_/K_2_TeO_3_-exposed *E. coli* AG1/pCA24N*gshA* pellets (Fig. 2A, 2 and 4, respectively). No fluorescence was observed in untreated *E. coli* AG1/pCA24N*gshA* or in cells exposed only to K_2_TeO_3_ (Fig. 2A, 1 and 3, respectively). As expected, AG1/pCA24N*gshB* cells did not produce fluorescent structures under any treatment (Fig. 2B). No fluorescence was observed in cell supernatants, suggesting that produced fluorescent structures are either inside cells or associated with the cell surface. Fluorescence of Cd^+2^-exposed cells most probably reflects CdS NPs, as has been previously described in cells expressing thiol-rich metal-binding peptides [11], [14].

### Metal Content Assessment of the Biosynthesized NPs

To evaluate metal content in fluorescent cells, ICP-AES elemental analysis of cell pellets was carried out. Results showed that in most conditions tested the AG1/pCA24N*gshA* strain displayed higher Cd and Te content than AG1/pCA24N*gshB* cells (Table 1). Interestingly, despite Te per cent increase in AG1/pCA24N*gshA* cells exposed to CdCl_2_/K_2_TeO_3_, no differences of intracellular Cd content were observed. Given that under these conditions cells do not display black deposits (Te^0^) this result suggests that fluorescence is probably a consequence of CdTe production. The ICP metal determinations clearly indicates that when *E. coli* wt, *gshA* or *gshB* strains were exposed to metals, only the fluorescent cells (*E. coli gshA*) display higher intracellular levels of Cd and Te as compared to none fluorescent cells (*E. coli* wt or *gshB*).

**Table 1:** Cd and Te content of *E. coli* expressing *gshA* or *gshB* genes.

	AG1/pCA24N*gshA*		AG1/pCA24N*gshB*	
Treatment	Cd	Te	Cd	Te
CdCl_2_	**3.7**	nd	0.69	bd
K_2_TeO_3_	bd	0.063	bd	bd
CdCl_2_/K_2_TeO_3_	**0.6**	**0.075**	0.69	0.035

Cd and Te content (%) was determined in fluorescent and non-fluorescent cells after metal exposure. Conditions in which fluorescent cells were observed are indicated in bold numbers; bd and nd stands for below detection limit and not determined, respectively.

### Fluorescence Microscopy of AG1/pCA24N*gshA* cells

This technique was used to further characterize bacterial cell fluorescence. Morphology of the AG1/pCA24N*gshA* strain exposed to the metals under study indicates that cells were highly stressed since filamentous and fragmented cells are observed after 24 h exposure. Epifluorescence microscopy revealed that fluorescence was not evenly distributed in the bacterial population and just some cells were fluorescent (Fig. 3A and B). Interestingly, fluorescence seems to accumulate in well defined structures which are found both inside cells and in the extracellular milieu (Fig. 3B). When exposed to UV light, cells displayed different fluorescence colors (green, yellow, red and blue, Fig. 3C), suggesting that CdTe QDs of different sizes and/or shapes are being produced and also that the biosynthesis process is not uniform under these experimental conditions.

### Purification and Characterization of Biosynthesized NPs

To demonstrate that the observed cell fluorescence is the consequence of CdTe QDs biosynthesis, these structures were purified and characterized. As shown in Figure 4A, concentrated cell suspensions of CdCl_2_- or CdCl_2_/K_2_TeO_3_-treated bacteria displayed fluorescence when exposed to UV light. Untreated cells did not display fluorescence and were used as control. After cell disruption, fluorescent structures were purified as described in Methods. When exposed to UV light, fractions from metal-exposed cells became fluorescent, as contrasted to those of control, untreated cells (Fig. 4B).

A Dynamic Light Scattering (DLS) analysis was carried out to confirm the presence of nano-sized material in fluorescent fractions. Fluorescent material purified from CdCl_2_- or CdCl_2_/K_2_TeO_3_-treated *E. coli* AG1/pCA24N*gshA* exhibited nanostructured material whose dimensions averaged 5.98 and 4.8 nm, respectively (Fig. 4C and D), which are in agreement with those reported for CdS and CdTe QDs, respectively [11], [28], [19], [29]. This size could also represent thiol- or GSH-capped QDs, since it falls in the size category of previously synthesized CdTe-GSH NPs [19], [29]. The DLS analysis also shows a wide size distribution of NPs (Fig. 4C and D), a result that is in agreement with the different colors of cells as determined by fluorescence microscopy (Fig. 3C). Additional size information for the CdTe sample was gathered through atomic force microscopy (Fig. S2); particle size was shown to be ∼2–3 nm, a result that is consistent with nanocrystal sizes reported by other authors [16], [17], [19], [29].

To confirm that these fluorescent NPs contain the expected metallic elements, an elemental analysis of purified samples was carried out. ICP-AES analysis of purified samples from cells exposed to either only CdCl_2_ or CdCl_2_/K_2_TeO_3_ indicated that fluorescence was related to the presence of Cd or Cd/Te, respectively (Table 2). In addition, the Cd/Te ratio (∼7) is close to that expected for CdTe NPs and is in agreement with those previously reported for CdTe-GSH QDs [19], [29].

**Table 2:** Elemental analysis of purified nano-sized structures.

NP	Cd	Te
Cd	5	bd
CdTe	7.13	0.98

Cd and Te content (ppm) in purified NPs from cells exposed only to CdCl_2_ (Cd) or CdCl_2_/K_2_TeO_3_ (CdTe) were determined as described in Methods; bd stands for below detection limit.

**Figure 5:**
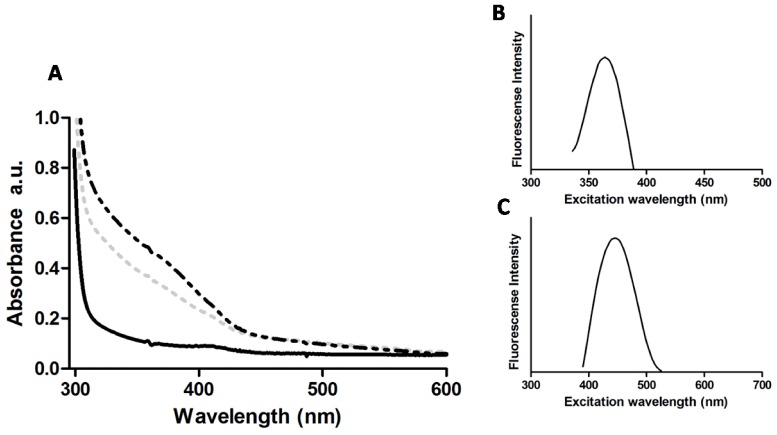
Absorbance and fluorescence spectra of purified NPs. **A**, absorption spectra of NPs from unexposed (solid black line), exposed to CdCl_2_ (dashed grey line) or CdCl_2_/K_2_TeO_3_ (dashed black line) cells. **B** and **C**, emission spectra of NPs from cells exposed to CdCl_2_ or CdCl_2_/K_2_TeO_3,_ respectively.

On the other hand and since they are well defined characteristics of QDs, absorption and emission spectra of purified samples were determined. A noticeable difference in the absorbance spectra (300–450 nm) was observed between cell treatments and untreated controls (Fig. 5A). Absorption spectra were those expected for biosynthesized QDs and are in agreement with those previously reported for CdS and CdTe QDs [11], [16], [17], [28].

Samples from CdCl_2_-exposed cells displayed a fluorescence maximum at 350–400 nm (Fig. 5B), characteristic of CdS QDs [11]. On the other hand, a peak above 450 nm was determined in the sample purified from cells exposed to CdCl_2_/K_2_TeO_3_ (Fig. 5C). This fluorescence peak corresponds to that expected for CdTe QDs [16], [17]. These spectroscopic properties indicate that CdS and CdTe NPs are being bacterially produced under these experimental conditions.

**Figure 6:**
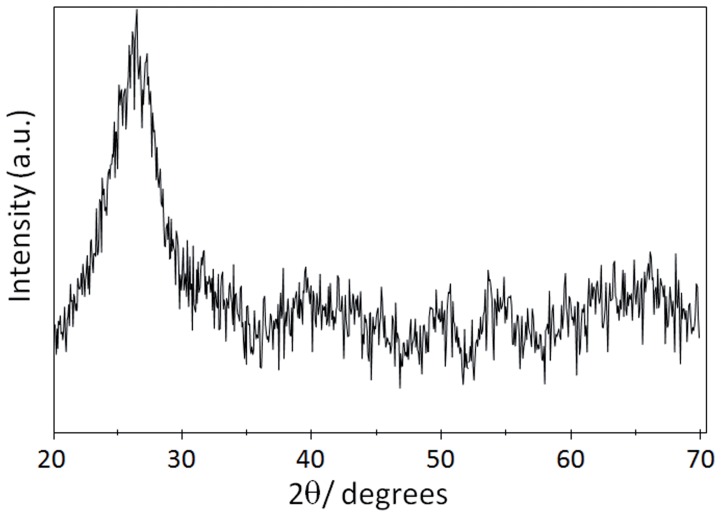
XRD pattern of biosynthesized CdTe QDs. CdTe NPs were purified from *E. coli gshA* cells as described in Methods and the presence of crystalline structures analyzed by XRD.

XRD analyses were performed to further confirm that the produced NPs are crystal-shaped. The XRD peaks were located between positions for a pure cubic CdTe crystal (Fig. 6) and for CdS greenockite crystals (not shown). The strong peak at 27,5° observed in biosynthesized CdTe NPs corresponds to the (111) planes of the standard pattern for cubic CdTe [30]. Altogether, XRD results indicate that biosynthesized QDs have a crystalline structure similar to those produced by chemical methods and particularly to biosynthesized CdTe QDs [30], [31], [32], [33].

**Figure 7:**
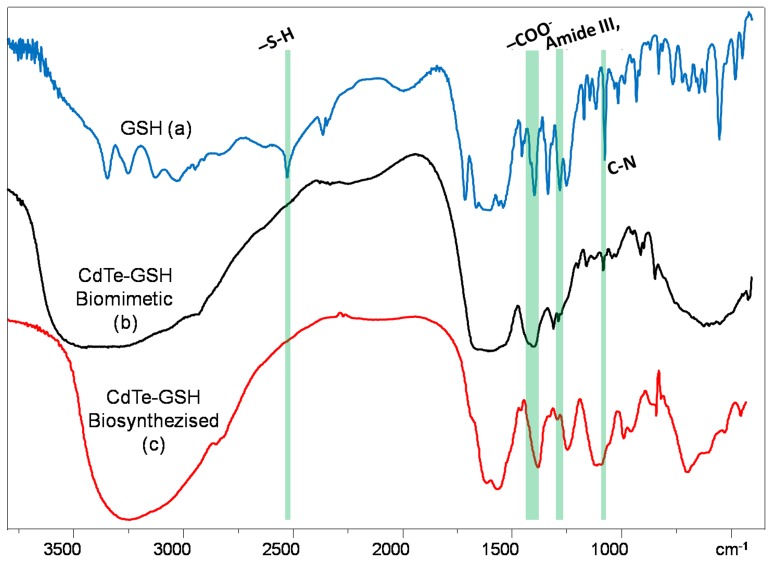
FTIR spectroscopy of GSH (A), chemically synthesized (B) and biosynthesized (C) CdTe-GSH QDs. Biological CdTe NPs where purified as described in Methods and chemical, biocompatible CdTe QDs where synthesized as described [19]. FTIR spectroscopy spectra were recorded and compared with a GSH standard.

On the other hand, to confirm that the biosynthesized NPs are GSH-capped as those previously produced by the biomimetic method [19], infrared spectra of GSH (a), GSH−capped CdTe QDs (b) and the biosynthesized CdTe QDs (c) were compared. Characteristic GSH absorption broad bands around 1713–1602 cm^−1^ (symmetric νCOO^−^), 1397 cm^−1^ (asymmetric νCOO^−^), 1713 cm^−1^ (νC = O) and 1260 cm^−1^ (δOH), indicating −COOH group are observed (Fig. 7a). Peaks around 3346–3030 cm^−1^ (symmetric νN-H) and 2526 cm^−1^ indicate -NH_2_ and -SH(νS-H) groups, respectively. In the spectra corresponding to CdTe NPs produced by both chemical and biosynthetic methods (Fig. 7b and 7c), disappearance of S-H group vibration 2526 cm^−1^ (νS-H) is clear and is likely a consequence of a covalent bond established between Cd and GSH. Furthermore, in biosynthesized CdTe NPs it is possible to observe other bands such as 1595 cm^−1^ (symmetric νCOO^−^), 1396 cm^−1^ (asymmetric νCOO^−^) and 1283 cm^−1^ (δOH) which indicate −COOH group and νC-N around 1120–1022 cm^−1^. All the observed bands are similar to those found in chemically produced CdTe-GSH NPs (Fig. 7b), confirming that our biosynthesized CdTe NPs are GSH-coated.

### Conditions Favoring QDs Biosynthesis

**Figure 8:**
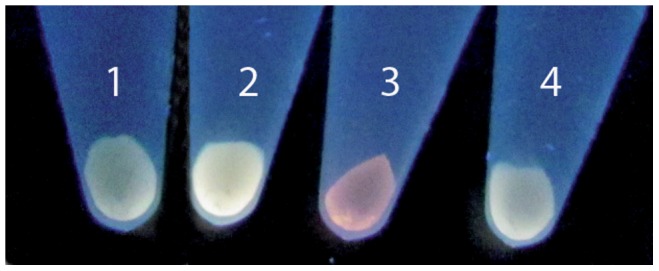
Effect of temperature, microaerophilic conditions and citrate on *E. coli* fluorescence. UV-exposed *E. coli* AG1/pCA24N*gshA* cells previously incubated under NP biosynthesis conditions with some modifications: 1, control (37°C); 2, increased incubation temperature (42°C); 3, reducing agent (citrate buffer pH 7.0); 4, microaerophilic conditions.

Conditions such as increased incubation temperature, microaerophilic environments and the use of reducing buffers (citrate), known to favor the chemical synthesis of CdTe QDs [19], were tested for their effect upon bacterial NPs biosynthesis. Fig. 8 shows that with increasing incubation temperature (42°C), the fluorescence of cell pellets is enhanced when exposed to UV light (compare 1 and 2). Interestingly, incubation of bacteria with citrate buffer changes fluorescence color, suggesting that NPs with different spectroscopic properties are produced (compare lane 1 and 3). This could be the consequence of size, shape and/or composition changes mediated by a specific cellular status or factors. On the other hand, microaerophilic conditions did not seem to favor or alter the cell fluorescence, as compared to controls (compare lane 1 and 4). Other environmental factors such as increased pH values seem to prevent NP formation and no fluorescence was observed when cells were incubated at basic pH (Fig. S3).

## Discussion

As a consequence of the multiple applications of nano-sized compounds, interest in manufacturing nanomaterials has grown exponentially during the last decade. Unfortunately, most NP-synthesizing procedures described to date are expensive, require dangerous compounds and produce NPs with elevated toxicity [34], [35]. In this context, an alternative procedure to increase NPs biocompatibility has been the use of non-toxic stabilizing agents that reduce the oxidant properties of NPs and increase their solubility in aqueous systems [36], [37]. Based on these considerations, a number of thiols as mercapto succinic acid (MSA), thioglycolic acid (TGA) and mercapto propionic acid (MPA), among others, have been successfully bound to QDs [31], [38]–[40]. Also, the use of biological thiols like GSH or cysteine as QDs-stabilizing agents has been reported [32], [33].

Recently, and to determine the minimal conditions for synthesizing CdTe QDs using biocompatible agents, we developed a biomimetic chemical synthesis procedure that requires only GSH as a reducing and stabilizing agent and Cd and Te salts as substrates [19]. This protocol functions under mild conditions of temperature, pH and requires substrates that are normally found in microorganisms such as GSH as well as compounds like CdCl_2_ and K_2_TeO_3_ whose effects in cells have been documented [19], [23], [41]. GSH plays a crucial role acting as Te^4+^to Te^2−^ reductant and, at the same time, stabilizing NPs by interacting with Cd^2+^to form a cap of oxidized thiol [19].

Most procedures for the biological synthesis of cadmium QDs such as CdS, CdSe or CdTe described to date use Cd^2+^salts as cadmium donors [11]–[15]. On the other hand, we believe that Te^2−^, required for CdTe biosynthesis, could be generated by GSH-mediated intracellular tellurite reduction. Moreover, cells exposed to tellurite become dark as consequence of Te^4+^-Te^0^ reduction, a phenomenon that is mediated -at least in part- by cellular thiols such as GSH [22] and enzymes as nitrate reductase, catalase and pyruvate dehydrogenase [42], [43], [44]. Although reduction of Te^4+^to Te^2−^ is an energetically unfavorable process, we propose that this chemical reduction can be improved by modifying culture conditions or manipulating the expression of defined genes. Evidence of this is the intracellular Te^2−^ generation observed during the evolution of methylated Te compounds such as (CH_3_)_2_Te in *E. coli* overexpressing the *Geobacillus stearothermophilus* V *ubiE* gene [45].

Based on the experimental conditions of the biomimetic protocol [19] and the known interactions of GSH with CdCl_2_ and K_2_TeO_3_, an *E. coli* strain with increased levels of GSH was used to biosynthesize CdTe QDs in the presence of CdCl_2_ and K_2_TeO_3_. The *E. coli* AG1/pCA24N*gshA* strain displayed higher RSH and GSH levels than the wt or AG1/pCA24N*gshB* strains (Fig. 1 A and B). Despite the fact that *gshA* and *gshB* genes are both involved in GSH biosynthesis and also because GshA catalyzes the final and limiting step in GSH synthesis, it was not surprising to find higher glutathione content in the *gshA*-expressing strain [25]. Thus, this strain represented an excellent candidate to test the role of GSH as reducing and stabilizing agent in the biological synthesis of CdTe QDs. As expected, after exposure to sub-lethal Cd and Te concentrations, cells overexpressing *gshA* displayed fluorescence under UV excitation (Fig. 2A). In contrast, no fluorescence was observed in wt or *gshB*-expressing cells; GSH is thus favoring the synthesis of fluorescent structures. Since fluorescence is an intrinsic QDs feature, this result represents the first evidence suggesting that QDs are being produced by bacteria (Fig. 2). Fluorescent cells displayed higher Cd and Te amounts (Table 1), a result that correlates fluorescence with the incorporation of metal substrates for QDs biosynthesis. This observation could be the basis for future applications in heavy metal bioremediation associated with the production of QDs for biotechnological purposes. In this context, preliminary experiments in our laboratory indicate that upon CdCl_2/_K_2_TeO_3_ exposure, other microorganisms like *Saccharomyces cerevisiae* and *Aeromonas caviae* ST produce fluorescent structures whose spectroscopic properties resemble that of CdTe QDs (not shown). This data also suggests that QDs biosynthesis seems to not be restricted to a particular species, thus facilitating putative biotechnological applications.

Recently, the extracellular synthesis of CdTe QDs using *E. coli* and *S. cerevisiae* was reported [16], [17]. CdTe NPs were produced in LB growth medium in the presence of MSA, CdCl_2_ and Na_2_TeO_3_, and require adding the strong chemical reducing agent NaBH_4_. Those authors proposed a mechanism in which microorganisms secrete some unidentified protein factor that allows the chemical synthesis of CdTe QDs in the extracellular milieu. Interestingly, we were able to synthesize our QDs under conditions similar to those described by Bao *et al*. (2010) but in the absence of bacterial cells, i.e. using only CdCl_2_, K_2_TeO_3_, NaBH_4_, LB medium and MSA. Highly fluorescent QDs were obtained in LB media (pH 7.0) in the presence of 4 mM CdCl_2_, 1 mM K_2_TeO_3_, 15 mM MSA and 10 mM NaBH_4_ after 2 and 3 h synthesis (Fig. S4). Nevertheless, extracellular CdTe QDs synthesis is still puzzling, and the mechanism(s) involved in the biosynthesis are unknown.

Fluorescence microscopy was the first approach to characterize the bacterially-produced fluorescent structures. Even though not all cells were fluorescent, those displaying fluorescence exhibited a common morphology with vesicles-like structures which can be found inside or outside cells (Fig. 4). When samples were excited at 330 or 488 nm a highly fluorescent spot was observed in these structures. Since a wide range of excitation wavelengths is another intrinsic property of CdTe QDs, these results suggest that fluorescent semiconductor nanostructures are being produced. A possibility is that these vesicle-like structures actually correspond to inclusion bodies where NPs are accumulated, which would be consequence of GshA overproduction or the metal-mediated stress (evidenced by cell damage and filamentation). In this context, the fluorescent structures described here resemble outer membrane vesicles that some Gram negative bacteria release in response to envelope stress [46].

This idea is supported by microarray studies performed by our group in *E. coli* exposed to nonlethal-tellurite concentrations (unpublished results). The induction of the stress envelope regulators RseA and YaeL, the murein-related *yiiX*, *ddl*, *lpxC,* and some genes related with plasma membrane proteins such as *nadH*, *narI narJ* and *ndH*, among others, suggests the generation of an envelope stress in these conditions. Experiments to unveil the nature of these vesicle-like structures are being carried out in our laboratory.

NPs were obtained by lysozyme treatment and sonic disruption of cells. However, fluorescent structures could not be recovered in the supernatants as has been reported for other QDs [11], [16], [17], [28], and SDS treatment was required to purify them. This suggests that NPs are being produced through different cellular processes in the AG1/pCA24N*gshA* strain, probably involving the association with the cell membrane or membrane-derived vesicles.

Elemental analysis confirmed that fractions enriched in fluorescent structures contain the expected metals (Table 2). Cells exposed to CdCl_2_ or CdCl_2_/K_2_TeO_3_ displayed a high content of Cd and Cd+Te, respectively, indicating that produced QDs correspond to CdS and CdTe. Particularly in the case of the CdTe fraction, the Cd/Te ratio was slightly higher than those reported for CdTe QDs produced by chemical methods [29]. Higher Cd content is probably the consequence of binding to capping agents which in this case would correspond to GSH (or cysteine), as has been previously described for CdTe-GSH QDs [19].

DLS analysis indicated that purified samples contained nano-sized material averaging 5.98 and 4.8 nm for CdS and CdTe QDs, respectively. The ∼5 nm size observed for CdTe QDs is within the dimensions expected for a population of QDs conformed mainly by red-colored NPs [19], [30]. However, size distribution in the case of both samples reflects that a variable population of NPs with sizes ranging from 4–8 and 3–6 nm for CdS and CdTe, respectively, are produced by the bacterial population. This observation is in agreement with fluorescence microscopy results, which showed cells of different colors when exposed to UV light (Fig. 3). Visualization of purified samples by AFM led to the conclusion that biosynthesized particles with uniform characteristics displayed 2–3 nm sizes in the CdTe sample. All size determination data and XRD analyss of purified samples indicate that CdTe NPs show characteristics of a pure cubic CdTe nanocrystal.

Besides nanometric size, one of the most noticeable QDs features is their special spectroscopic properties. CdS QDs absorb and emit light in the blue zone while CdTe QDs shift their absorption to the red zone of the spectrum [11], [16], [17], [28]. Biosynthesized NPs displayed increased absorption between 300 and 500 nm, similar to that previously reported for biosynthesized cadmium QDs [11], [16], [17], [28]. As expected, a slight absorbance red shift was determined for CdTe QDs (Fig. 5A). Differences observed between biosynthesized and chemically-produced QDs are probably the consequence of light-absorbing biomolecules.

Fluorescence spectra of CdS NPs exhibited a peak near 370 nm, which is in agreement with that reported for CdS QDs produced by cells or chemical protocols [11], [34]. In addition, the peak width was ∼80 nm, indicating the presence of different-sized NPs. On the other hand, emission spectra of purified NPs from CdCl_2_/K_2_TeO_3_-exposed cells showed a peak at 450 nm, characteristic of CdTe QDs [16], [17]. In this case the peak width was ∼110 nm, again confirming the presence of different-sized NPs.

Once the production of QDs by *E. coli* was confirmed, the effect of microaerophilia, culture temperature or the use of reducing buffers on cell fluorescence was investigated. Despite the fact that it has been reported that cellular reduction of Te^4+^is favored by O_2_ [43], no effect on cell fluorescence was observed under oxygen deprivation (Fig. 8). On the other hand, slightly higher temperatures improved cell fluorescence upon metal exposure, confirming the importance of this parameter in CdTe QDs production as shown in the biomimetic method [19]. Fluorescence color was shifted to red when citrate buffer was used, indicating that different NPs are being produced. These observations suggest that little changes in culture conditions can alter NPs properties, opening the opportunity to evaluate new conditions or microorganisms able to produce QDs with desired properties, as color, size and bound molecules. For instance, the effect of temperature offers the chance of using thermophiles for improved biological production of semiconductor NPs. Despite all these observations, optimal conditions and factors to enhance NP biosynthesis by microorganisms is far from being understood.

Finally, results from this work indicate that thiol content and redox state can promote the formation of bimetallic CdTe QDs after treating *E. coli* with cadmium and tellurium salts. Microorganisms displaying high-reducing environments and/or increased antioxidant defenses as GSH or RSH, represent excellent candidates to develop cell nano-factories for synthesizing different QDs.

## Materials and Methods

### Bacterial Strains and Culture Conditions

The *E. coli* strains AG1 (wild type) and those over expressing the homologous *gshA* (encoding L-glutamate cysteine ligase) or *gshB* (encoding glutathione synthetase) genes were from the ASKA collection [47]. *E. coli* AG1 is the parental strain of those over expressing *gshA* and *gshB* and all of them have the same genetic background.

Cells were routinely grown at 37°C in Luria-Bertani (LB) medium with shaking. When required, chloramphenicol (25 µg/ml) was added to the medium. Growth in liquid medium was normally started with 1/100 dilutions of overnight cultures. Induction of *gshA* and *gshB* genes was carried out in the presence of 0.5 mM isopropil-β-D-1-thiogalactopyranoside (IPTG).

### Quantification of Intracellular GSH and RSH

Cells were grown to OD_600_ ∼0.5 and IPTG was added. After inducing for 4 or 16 h, 1 ml aliquots were collected and cells were washed twice with PBS buffer and suspended in 100 µl of 5% sulfosalicylic acid. Cells were disrupted by repeated cycles of freeze/thawing and after centrifuging at 10,000×*g* for 15 min supernatants were saved and used for RSH determination.

Ellman’s reagent [5,5′-dithiobis (2-nitrobenzoic acid) or DTNB] was used for quantifying reduced thiols [48]. DTNB reacts with GSH to form 5-thionitrobenzoic acid (TNB) and GS-TNB. Absorbance at 412 nm was determined after 5 min at 37°C. Calibration curves were constructed using GSH solutions of known concentration. Intracellular GSH was determined according to the protocol previously described by Allen *et al*. [49].

### Nanoparticle Biosynthesis

*E. coli* AG1 harboring plasmid pCA24N*gshA* was grown to OD_600_∼0.5, 500 µM IPTG was added and after inducing for 4 h, cells were exposed to 54 µM CdCl_2_ and 2 µM K_2_TeO_3_ for 24 h. After washing twice with 50 mM potassium phosphate pH 7.4 buffer, cells were centrifuged for 15 min at 13.000×*g* and stored at −80°C until use.

Microaerophilic conditions were obtained by filling culture tubes completely with sterile mineral oil at the top to avoid oxygenation. For high temperature or reducing buffer conditions, cultures were incubated at 42°C or suspended in 50 mM citrate buffer pH 7.0, respectively, immediately after metal addition.

### Fluorescence Microscopy

An aliquot of the fluorescent AG1/pCA24N*gshA* culture was diluted and mounted on a glass slide with 2.5% 1,4-diazobiciclo [2,2,2] octane (DABCO) and glycerol. Samples were assessed for individual cell fluorescence using an Olympus BX51 fluorescence microscope following excitation at 350 nm.

Monochromatic Epifluorescence microscopy (Olympus, model CKX41) was used to identify cell structures and morphology in NP-producing bacteria. Cells were fixed with PBS-4% paraformaldehyde for 30 min at 25°C and washed three times with PBS. Samples were mounted onto slides with 10% Mowiol−2.5% DABCO and visualized using an UV-DAPI/FITC filter (Ex 400–420 nm, Em 448–465 nm/Ex 475–500 nm, Em 510–560). Images were processed with MIS Viewer F 3.0 software.

### Purification of Biosynthesized Nanoparticles

Cells were suspended in 1 ml of PBS buffer containing 1 mg/ml lysozyme and incubated at room temperature for 30 min. After centrifuging at 5,000×*g* for 5 min cells were disrupted by sonication (3 min with 30 s intervals). The fluorescent debris was treated with 4% SDS at 90°C for 30 min and incubated overnight at 37°C to solubilize membranes. Afterwards, 1 ml of the solution was ultracentrifuged over a 40–60% sucrose cushion at 300,000×*g* for 2 h. The fluorescent fraction was detected by exposure to UV light (312 nm) and concentrated using a 30 kDa cut-off membrane. The resulting solution was heated at 90°C for 20 min and centrifuged to remove contaminating proteins. This solution, highly enriched in NPs, was used in NPs characterization experiments.

### Nanoparticle Characterization

i) *Absorption and fluorescence spectroscopy*: to get absorbance and fluorescence spectra, purified NPs were diluted 1/10 with ultrapure water. Absorbance spectra were recorded with a Perkin-Elmer Lambda 11 UV-vis spectrophotometer using MiliQ water as blank. Fluorescence spectra were obtained with an ISS-PC photocounting spectrofluorimeter. An excitation spectra was constructed recording fluorescence emission at 515 nm while exciting the sample at different wavelengths (300–500 nm); ii) *Dynamic light scattering (DLS)*: dynamic light scattering of purified fluorescent fractions was used to evaluate NP size as described before [19]. Data were acquired using a Zetasizer nano S90 (Malvern Instruments Limited, UK) instrument with a refraction index of 2.6 and 4-optical sides disposable cuvettes; iii) *Atomic force microscopy (AFM):* AFM measurements were conducted in a NT-MDT NTEGRA prima equipment using silica-etched tips. Typical samples were investigated at scan rates of 1–3 Hz with 256×256 pixel resolution during image capturing. Samples were dropped onto a mica sheet. Size determination was performed individually recording the z-axis height of each particle and subtracting the surrounding z-axis mica sheet height; iv)*Elemental analysis by inductive coupled plasma atomic emission spectrometry*: determining and quantifying Cd and Te metal species in both cell pellets and purified NPs was carried out using Spectro CIROS Vision ICP-AES. Strains pCA24N*gshA* and pCA24N*gshB* were grown as described and treated with CdCl_2_, K_2_TeO_3_ or both for 24 h at 37°C.

After centrifuging at 10,000 *g* for 5 min, samples were washed twice with sterile Millipure water, heated and taken to dryness with concentrated HNO_3_ and then dissolved in 10 mL of 10% nitric acid. Cd and Te content was determined using calibration curves specifically constructed for each element and normalized to the dry bacterial mass. The Te analytical line was 214.281 nm and for Cd, 228.802 nm; v) *X-ray diffraction analysis:* Power X-ray diffraction (XRD) measurements were performed using a Diffractometer Bruker D8 Advance using Cu Ka radiation. For XRD characterization, an aqueous QDs solution was diluted with 1 volume of ethanol and centrifuged at 4,000 rpm for 10 min. The precipitate was collected, dropped onto a glass slide and dried at room temperature. vi) *IR studies*: Infrared transmission spectra of purified NPs were recorded in KBr pellets from 2000 to 400 cm^−1^ on a Nicolet Impact 410 IR spectrophotometer.

### Statistical Analysis

Statistical analysis was carried out using the GraphPad Prism v5.0 software. One-way ANOVA was used for establishing significant differences between more than two groups using a p<0.05. Dunnet post-test was used to compare differences between all the groups (GSH quantification) and Bonferroni post-test to compare different samples with the control (RSH determination).

## Supporting Information

Figure S1 **GshA and GshB induction kinetics.** SDS-PAGE analysis of total proteins from *E. coli* AG1 and *E. coli gshA* after IPTG (0.5 mM) treatment for the indicated times.
(TIF)

Figure S2 **AFM of biosynthesized CdTe QDs.** Biologically synthesized CdTe nanoparticles were purified as described in Methods and the nanometric size was evaluated by AFM.
(TIF)

Figure S3 **Effect of pH on nanoparticle biosynthesis.** *E. coli gshA* were grown to stationary phase and suspended in water or phosphate buffer adjusted to different pH values. Cells were exposed to both CdCl_2_ and K_2_TeO_3_ in (left to rigth): water or phosphate buffer pH 7.0, 8.0, 9.0, 10.0 and 11.O.
(TIF)

Figure S4 **Synthesis of CdTe QDs in bacterial growth media.** Fluorescence spectra of CdTe QDs synthesized in LB media, pH 7.0, amended with 4 mM CdCl_2_, 1 mM K_2_TeO_3_, 15 mM MSA and 10 mM NaBH_4_ at the indicated time intervals.
(TIF)

Table S1 **Bacterial strains used in this work.**
(DOC)

## References

[1] Bang J, Kamat P (2009) Quantum dot sensitized solar cells. A tale of two semiconductor nanocrystals: CdSe and CdTe. ACS Nano 3: 1467–1476. 19435373 10.1021/nn900324q

[2] Azzazy H, Mansour M, Kazmierczak S (2007) From diagnostics to therapy: prospects of quantum dots. Clin Biochem 40: 917–927. 17689518 10.1016/j.clinbiochem.2007.05.018

[3] Mandal D, Bolander ME, Mukhopadhyay D, Sarkar G, Mukherjee P (2006) The use of microorganisms for the formation of metal nanoparticles and their application. Appl Microbiol Biotechnol 69: 485–492. 16317546 10.1007/s00253-005-0179-3

[4] Ahmad A, Senapati S, Khan M, Kumar R, Ramani R, et al (2003) Intracellular synthesis of gold nanoparticles by a novel alkalotolerant actinomycete, *Rhodococcus* species. Nanotechnology 14: 824–828.

[5] Klaus T, Joerger R, Olsson E, Granqvist CG (1999) Silver-based crystalline nanoparticles, microbially fabricated. Proc Natl Acad Sci USA 96: 13611–13614. 10570120 10.1073/pnas.96.24.13611 PMC24112

[6] Bharde AA, Parikh RY, Baidakova M, Jouen S, Hannoyer B, et al (2008) Bacteria-mediated precursor-dependent biosynthesis of superparamagnetic iron oxide and iron sulfide nanoparticles. Langmuir 24: 5787–5794. 18454562 10.1021/la704019p

[7] Schüler D (2008) Genetics and cell biology of magnetosome formation in magnetotactic bacteria. FEMS Microbiol Rev 32: 654–672. 18537832 10.1111/j.1574-6976.2008.00116.x

[8] Matsunaga T, Suzuki T, Tanaka M, Arakaki A (2007) Molecular analysis of magnetotactic bacteria and development of functional bacterial magnetic particles for nano-biotechnology. Trends Biotechnol 25: 182–188. 17306901 10.1016/j.tibtech.2007.02.002

[9] Shankar S, Raj A, Ankamwar B, Singh A, Ahmad A, Sastry M (2004) Biological synthesis of triangular gold nanoparticles. Nat Mater 3: 482–488. 15208703 10.1038/nmat1152

[10] Ramanathan R, O’Mullane A, Parikh R, Smooker P, Bhargava S, Bansal V (2011) Bacterial kinetics-controllled shape-directed biosynthesis of silver nanoplates using *Morganella psychrotolerans* . Langmuir 27: 714–719. 21142094 10.1021/la1036162

[11] Kang S, Bozhilov K, Myung N, Mulchandani A, Chen W (2008) Microbial synthesis of CdS nanocrystals in genetically engineered *E. coli* . Angewan Chem Int Ed Eng 47: 5186–5189. 10.1002/anie.200705806 18512860

[12] Kowshik M, Deshmukh N, Vogel W, Urban J, Kulkarni SK, et al (2002) Microbial synthesis of semiconductor CdS nanoparticles, their characterization, and their use in the fabrication of an ideal diode. Biotechnol Bioeng 78: 583–588. 12115128 10.1002/bit.10233

[13] Kumar S, Ansary A, Ahmad A, Khan M (2007) Extracellular biosynthesis of CdSe quantum dots by the fungus, *Fusarium oxysporum* . J Biomed Nanotechnol 3: 190–194.

[14] Cui R, Liu H, Xie H, Zhang Z, Yang Y, et al (2009) Living yeast cells as a controllable biosynthesizer for fluorescent quantum dots. Adv Funct Mater 19: 2359–2364.

[15] Mi C, Wang Y, Zhang J, Huang H, Xu L, et al (2011) Biosynthesis and characterization of CdS quantum dots in genetically engineered *Escherichia coli* . J Biotechnol 153: 125–132. 21458508 10.1016/j.jbiotec.2011.03.014 PMC3102602

[16] Bao H, Hao N, Yang Y, Zhao D (2010a) Biosynthesis of biocompatible cadmium telluride quantum dots using yeast cells. Nano Res 3: 481–489.

[17] Bao H, Lu Z, Cui X, Qiao Y, Guo J, Anderson J, et al (2010b) Extracellular microbial synthesis of biocompatible CdTe quantum dots. Acta Biomater 6: 3534–3541. 20350621 10.1016/j.actbio.2010.03.030

[18] Park T, Lee S, Heo N, Seo T (2010) In vivo synthesis of diverse metal nanoparticles by recombinant *Escherichia coli.* . Angew Chem Int Ed 49: 7019–7024. 10.1002/anie.201001524 20842627

[19] Pérez-Donoso JM, Monrás JP, Bravo D, Aguirre A, Quest AF, et al. (2012) Biomimetic, mild chemical synthesis of CdTe-GSH quantum dots with improved biocompatibility. *PLoS ONE* doi: 10.1371/journal.pone.0030741. 10.1371/journal.pone.0030741 PMC3264638 22292028

[20] Schafer F, Buettner G (2001) Redox environment of the cell as viewed through the redox state of the glutathione disulfide/glutathione couple. Free Rad Biol Med 30: 1191–1212. 11368918 10.1016/s0891-5849(01)00480-4

[21] Helbig K, Bleuel C, Krauss G, Nies D (2008) Glutathione and transition-metal homeostasis in *Escherichia coli* . J Bacteriol 190: 5431–5438. 18539744 10.1128/JB.00271-08 PMC2493246

[22] Turner R, Weiner J, Taylor D (1999) Tellurite-mediated thiol oxidation in *Escherichia coli* . Microbiology 145: 2549–2557. 10517608 10.1099/00221287-145-9-2549

[23] Pérez J, Pradenas G, Navarro C, Henríquez D, Pichuantes S, et al (2006) *Geobacillus stearothermophilus* LV *cadA* gene mediates resistance to cadmium, lead and zinc in *zntA* mutants of *Salmonella enterica* serovar Typhimurium. Biol Res 39: 661–668. 17657347 10.4067/s0716-97602006000500009

[24] Pérez J, Calderón I, Arenas F, Fuentes D, Pradenas G, et al (2007) Bacterial toxicity of potassium tellurite: unveiling an ancient enigma. *PLoS ONE* 2: e211. 17299591 10.1371/journal.pone.0000211 PMC1784070

[25] Murata K, Kimura A (1982) Cloning of a gene responsible for the biosynthesis of glutathione in *Escherichia coli* B. Appl Environ Microbiol. 44: 1444–1448. 10.1128/aem.44.6.1444-1448.1982 PMC242208 6130744

[26] Gushima H, Yasuda S, Soeda E, Yokota M, Kondo M, et al (1984) Complete nucleotide sequence of the *E. coli* glutathione synthetase *gsh-II* . Nucleic Acids Res 12: 9299–9307. 6393055 10.1093/nar/12.24.9299 PMC320462

[27] Labrenz M, Druschel GK, Thomsen-Ebert T, Gilbert B, Welch SA, et al (2000) Formation of sphalerite (ZnS) deposits in natural biofilms of sulfate-reducing bacteria. Science 290: 1744–1747. 11099408 10.1126/science.290.5497.1744

[28] Sweeney R, Mao C, Gao X, Burt J, Belcher A (2004) Bacterial biosynthesis of cadmium sulfide nanocrystals. Chem Biol 11: 1553–1559. 15556006 10.1016/j.chembiol.2004.08.022

[29] Zheng Y, Gao S, Ying J (2007) Synthesis and cell-imaging applications of glutathione-capped CdTe quantum dots. Adv Mater 19: 376–380.

[30] Gaponik N, Talapin D, Rogach A, Hoppe K, Shevchenko E, et al (2002) Thiol-capping of CdTe nanocrystals: an alternative to organometallic synthetic routes. J Phys Chem B 106: 7177–7185.

[31] Qian H, Dong C, Weng J, Ren J (2006) Facile one-pot synthesis of luminescent, water-soluble, and biocompatible glutathione-coated CdTe nanocrystals. Small 6: 747–751. 10.1002/smll.200500530 17193117

[32] Ying E, Li D, Guo S, Dong S, Wang J (2008) Synthesis and bio-imaging application of highly luminescent mercaptosuccinic acid-coated CdTe nanocrystals. *PLoS ONE* 3: e2222. 18493618 10.1371/journal.pone.0002222 PMC2377334

[33] Bao H, Wang E, Dong S (2006) One-pot synthesis of CdTe nanocrystals and shape control of luminescent CdTe-cystine nanocomposites. Small 2: 476–480. 17193069 10.1002/smll.200500346

[34] Peng Z, Peng × (2001) Formation of high-quality CdTe, CdSe, and CdS nanocrystals using CdO as precursor. J Am Chem Soc 123: 183–184. 11273619 10.1021/ja003633m

[35] Hardman R (2006) A toxicologic review of quantum dots: Toxicity depends on physicochemical and environmental factors. Environ Health Perspect 114: 165–172. 16451849 10.1289/ehp.8284 PMC1367826

[36] Hoshino A, Manabe N, Fujioka K, Suzuki K, Yasuhara M, et al (2007) Use of fluorescent quantum dot bioconjugates for cellular imaging of immune cells, cell organelle labeling, and nanomedicine: surface modification regulates biological function, including cytotoxicity. J Artif Organs 10: 149–157. 17846713 10.1007/s10047-007-0379-y

[37] Schneider R, Wolpert C, Guilloteau H, Balan L, Lambert J, et al (2009) The exposure of bacteria to CdTe-core quantum dots: the importance of surface chemistry on cytotoxicity. Nanotechnology 20: 225101. 19433881 10.1088/0957-4484/20/22/225101

[38] Mandal A, Tamai N (2008) Inﬂuence of acid on luminescence properties of thioglycolic acid-capped CdTe quantum dots. J Phys Chem C 112: 8244–8250.

[39] Tian J, Liu R, Zhao Y, Xu Q, Zhao S (2009) Controllable synthesis and cell-imaging studies on CdTe quantum dots together capped by glutathione and thioglycolic acid. J Colloid Inter Sci 336: 504–509. 10.1016/j.jcis.2009.04.064 19476949

[40] Liu Y, Chen W, Joly AG, Wang Y, Pope C, et al (2006) Comparison of water-soluble CdTe nanoparticles synthesized in air and in nitrogen. J Phys Chem B 110: 16992–17000. 16927992 10.1021/jp063085k

[41] Chasteen T, Fuentes D, Tantaleán J, Vásquez C (2009) Tellurite: history, oxidative stress, and molecular mechanisms of resistance. FEMS Microbiol Rev 33: 820–832. 19368559 10.1111/j.1574-6976.2009.00177.x

[42] Avazéri C, Turner R, Pommier J, Weiner J, Giordano G, et al (1997) Tellurite reductase activity of nitrate reductase is responsible for the basal resistance of *Escherichia coli* to tellurite. Microbiology 143: 1181–1189. 9141681 10.1099/00221287-143-4-1181

[43] Calderón I, Arenas F, Pérez J, Fuentes D, Araya M, et al (2006) Catalases are NAD(P)H-dependent tellurite reductases. *PLoS ONE* 1: e70. 17183702 10.1371/journal.pone.0000070 PMC1762332

[44] Castro ME, Molina RC, Díaz WA, Pradenas GA, Vásquez CC (2009) Expression of *Aeromonas caviae* ST pyruvate dehydrogenase complex components mediate tellurite resistance in *Escherichia coli* . Biochem Biophys Res Commun 380: 148–152. 19168030 10.1016/j.bbrc.2009.01.078

[45] Araya MA, Swearingen JW, Plishker MF, Saavedra CP, Chasteen TG, et al (2004) *Geobacillus stearothermophilus* V *ubiE* gene product is involved in the evolution of dimethyl telluride in *Escherichia coli* K-12 cultures amended with potassium tellurate but not with potassium tellurite. J Biol Inorg Chem 9: 609–615. 15164269 10.1007/s00775-004-0554-z

[46] McBroom AJ, Kuehn MJ (2007) Release of outer membrane vesicles by Gram-negative bacteria is a novel envelope stress response. Mol Microbiol 63: 545–558. 17163978 10.1111/j.1365-2958.2006.05522.x PMC1868505

[47] Kitagawa M, Ara T, Arifuzzaman M, Ioka-Nakamichi T, Inamoto E, et al (2005) Complete set of ORF clones of *Escherichia coli* ASKA library (a complete set of *E. coli* K-12 ORF archive): unique resources for biological research. DNA Res 12: 291–299. 16769691 10.1093/dnares/dsi012

[48] Ellman G (1959) Tissue sulfhydril groups. Arch Biochem Biophys 82: 70–77. 13650640 10.1016/0003-9861(59)90090-6

[49] Allen S, Shea JM, Felmet T, Gadra J, Dehn PF (2001) A kinetic microassay for glutathione in cells plated on 96-well microtiter plates. Meth Cell Sci 22: 305–312. 10.1023/a:1017585308255 11549943

