# Supplementary figures and images for: Enhanced Glutathione Content Allows the In Vivo Synthesis of Fluorescent CdTe Nanoparticles by Escherichia coli
Source: PLoS One. 2012 Nov 21;7(11):e48657. doi: 10.1371/journal.pone.0048657 (PMC3504078; doi:10.1371/journal.pone.0048657)

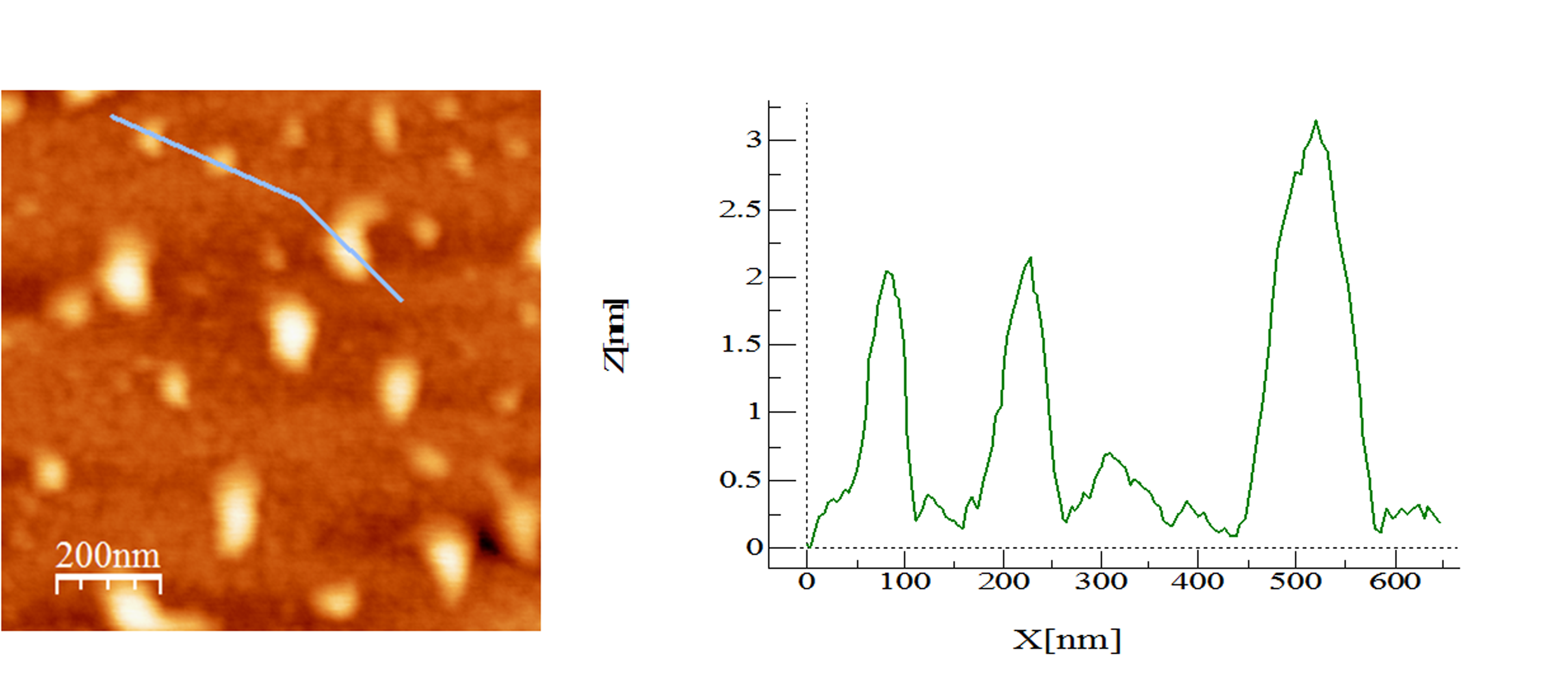

Supplement: Figure S2 — AFM of biosynthesized CdTe QDs. Biologically synthesized CdTe nanoparticles were purified as described in Methods and the nanometric size was evaluated by AFM. (TIF) [file pone.0048657.s002.tif]

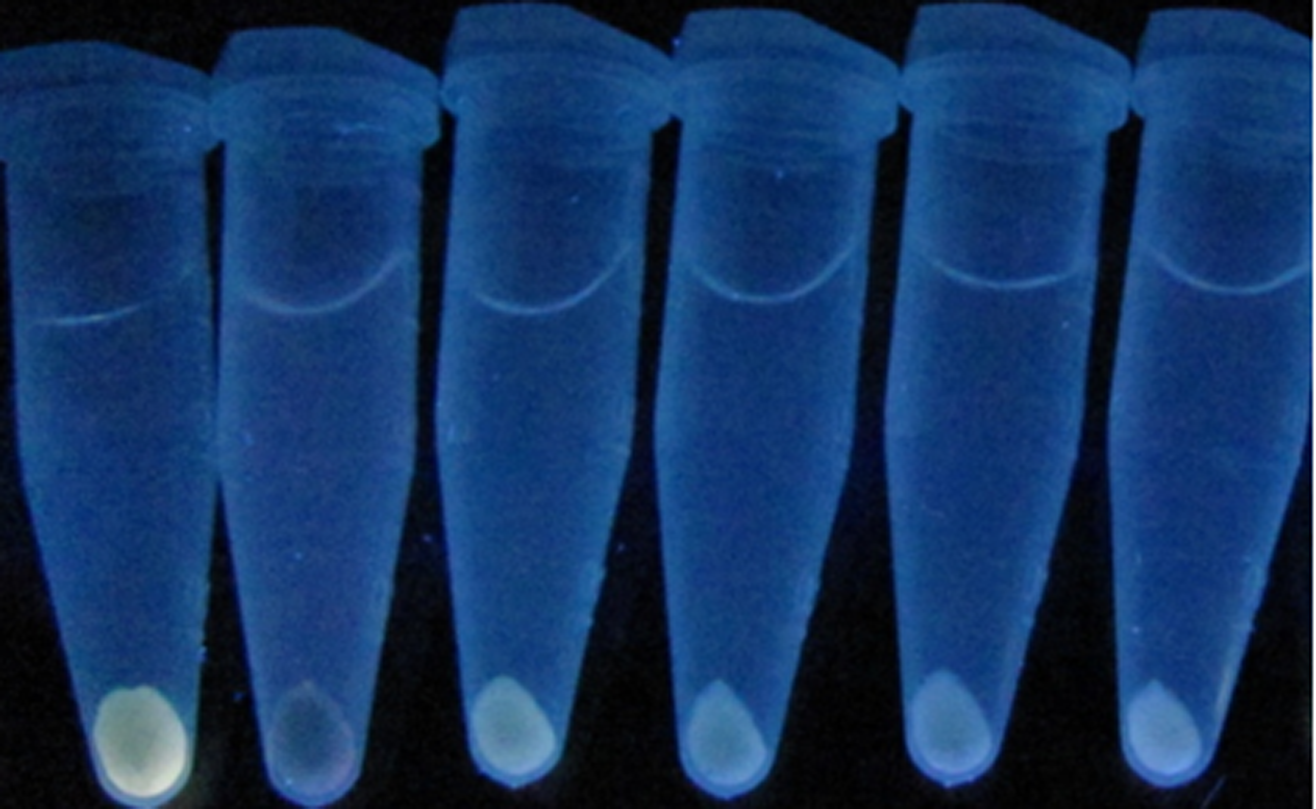

Supplement: Figure S3 — Effect of pH on nanoparticle biosynthesis. E. coli gshA were grown to stationary phase and suspended in water or phosphate buffer adjusted to different pH values. Cells were exposed to both CdCl2 and K2TeO3 in (left to rigth): water or phosphate buffer pH 7.0, 8.0, 9.0, 10.0 and 11.O. (TIF) [file pone.0048657.s003.tif]

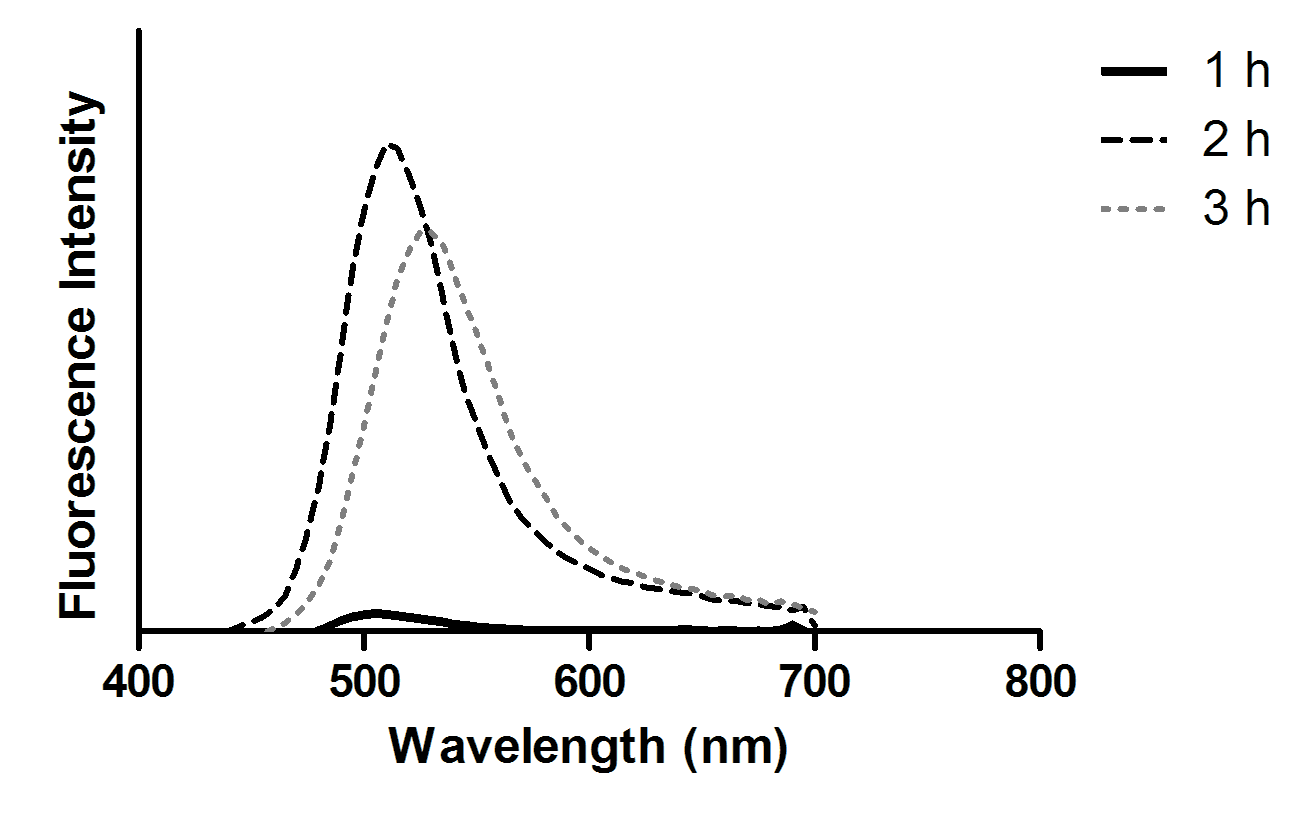

Supplement: Figure S4 — Synthesis of CdTe QDs in bacterial growth media. Fluorescence spectra of CdTe QDs synthesized in LB media, pH 7.0, amended with 4 mM CdCl2, 1 mM K2TeO3, 15 mM MSA and 10 mM NaBH4 at the indicated time intervals. (TIF) [file pone.0048657.s004.tif]
