# Supplementary material for: Enhanced Glutathione Content Allows the In Vivo Synthesis of Fluorescent CdTe Nanoparticles by Escherichia coli
Source: PLoS One. 2012 Nov 21;7(11):e48657. doi: 10.1371/journal.pone.0048657 (PMC3504078; doi:10.1371/journal.pone.0048657)
Supplement: Table S1 — Bacterial strains used in this work. (DOC) [file pone.0048657.s005.doc]

**Table S1.** Bacterial strains used in this work.

| *E. coli* | Relevant genotype | Feature |
| --- | --- | --- |
| AG1 | *recA*1 *endA*1 *gyrA*96 *thi*-1 *hsdR*17(*r*K_*m*Kþ) *supE*44 *relA*1 | Wild type. Carries plasmid pCA24N |
| AG1/pCA24N*gshA* | AG1 harboring plasmid pCA24N*gshA* | *gshA* over expression |
| AG1/pCA24N*gshB* | AG1 harboring plasmid pCA24N*gshB* | *gshB* over expression |
